# Efficacy of hyperthermic intraperitoneal chemotherapy (HIPEC) in the management of malignant ascites

**DOI:** 10.1186/s12957-020-01956-y

**Published:** 2020-07-22

**Authors:** Jie Jiao, Chengzhen Li, Guanying Yu, Lei Zhang, Xiaoyan Shi, Jingdu Yan, Houjun Zhang, Peiming Guo

**Affiliations:** Department II of Gastrointestinal Surgery, Jinan Central Hospital, Cheeloo College of Medicine, Shandong University, Jinan, 250013 Shandong China

**Keywords:** Efficacy, Malignant ascites, Intraperitoneal hyperthermic chemotherapy

## Abstract

**Objective:**

The purpose of this study is to compare the difference of clinical efficacy between conventional intraperitoneal chemotherapy and HIPEC, so as to explore the clinical application value and advantages of HIPEC.

**Design:**

A retrospective analysis was conducted on 80 patients with malignant ascites admitted to our hospital from June 2017 to June 2019. The general clinical data and qualitative data of the treatment results of 80 patients with malignant ascites were processed by SPSS19.0 using *χ*^2^ test, and quantitative data were processed by *t* test. *P* < 0.05, statistical data can be considered statistically significant.

**Results:**

1. There was no significant change in vital signs and temperature in the observation group during the treatment, and the difference was not statistically significant.
2. The short-term total effective rate of patients in the observation group was 91.11%, and the short-term total effective rate of the patients in the control group was 40%.
3. There was no significant difference in the incidence of adverse reactions between the two groups of patients.

**Conclusion:**

Intraperitoneal hyperthermic chemotherapy combined with intravenous chemotherapy can significantly control malignant ascites and has small adverse reactions, which is worthy of clinical promotion and application.

## Introduction

Abdominal or systemic malignant tumors can easily induce diffuse lesion of the abdominal visceral wall and further lead to abnormal increase of peritoneal effusion, which is called malignant ascites clinically [1]. Clinical studies have found that malignant ascites develops rapidly, and patients without effective intervention may experience symptoms such as abdominal distention, abdominal pain, and even breathing difficulties [2]. Some studies have pointed out that the prognosis of patients with malignant ascites is very poor, the survival period of patients is short, the 5-year survival rate is less than 35% [3]. In recent years, hyperthermic intraperitoneal chemotherapy (HIPEC) is an effective method for auxiliary abdominal malignant tumor treatment, than traditional chemotherapy agents, in the treatment, and prevention of malignant tumors of peritoneal planting has extremely obvious clinical advantages [4]. Therefore, a retrospective study was conducted on 80 patients with malignant ascites treated in our hospital to compare the difference of clinical efficacy between conventional intraperitoneal chemotherapy and HIPEC, so as to explore the clinical application value and advantages of HIPEC.

## Material methods

### Inclusion and exclusion criteria

The inclusion criteria were as follows: (1) 16 ≤ age ≤ 85 years; (2) CT, MRI, exploratory laparotomy and/or intraperitoneal free cancer cells confirmed diffuse intraperitoneal implantation metastasis of tumor; and (3) ultrasonography confirmed the abdominal volume of > 3000 ml.

The exclusion criteria were as follows: (1) no malignant ascites or abdominal volume < 1500 ml, (2) extensive adhesion in the abdominal cavity, (3) inclusional peritoneal effusion, (4) total intestinal obstruction, (5) severe coagulation dysfunction, and (6) too old and obviously poor physical function.

### General case information

The 80 patients admitted to our hospital from June 2017 to June 2019 were divided into an observation group and control group according to different treatment methods. Among the 45 patients in the observation group, 25 were male and 20 were female. The mean age was 57.87 ± 9.733 years. Mean abdominal volume was 4353.3 ± 685.180 ml. There were 8 cases of primary appendiceal, 12 cases of primary gastric cancer, 20 cases of colon cancer, 2 cases of mesothelioma, and 3 cases of ovarian cancer. Among the 35 patients in the control group, there were 18 males and 17 females. The mean age was 58.0 ± 8.570 years. The mean abdominal volume was 4170.0 ± 646.55 ml. There were 6 cases of primary appendiceal, 10 cases of primary gastric cancer, 18 cases of colon cancer, none of mesothelioma, and 1 case of ovarian cancer. There were no statistically significant differences in gender, age, abdominal volume, and primary disease between the two groups (*P* > 0.05) (Table 1).

**Table 1:** Patient and procedure characteristics

Characteristic	Observation group	Control group	*P* value
Gender			
Male	25	18	0.822
Female	20	17	
Age (years)	57.87 ± 9.733	58.0 ± 8.570	0.760
Ascites volume (ml)	4353.3 ± 685.180	4170.0 ± 646.55	0.960
Type of primary disease			
Appendiceal	8	6	0.670
Colorectal	20	18	
Gastric	12	10	
Mesothelioma	2	0	
Ovarian	3	1	

### Methods

**Fig. 1:**
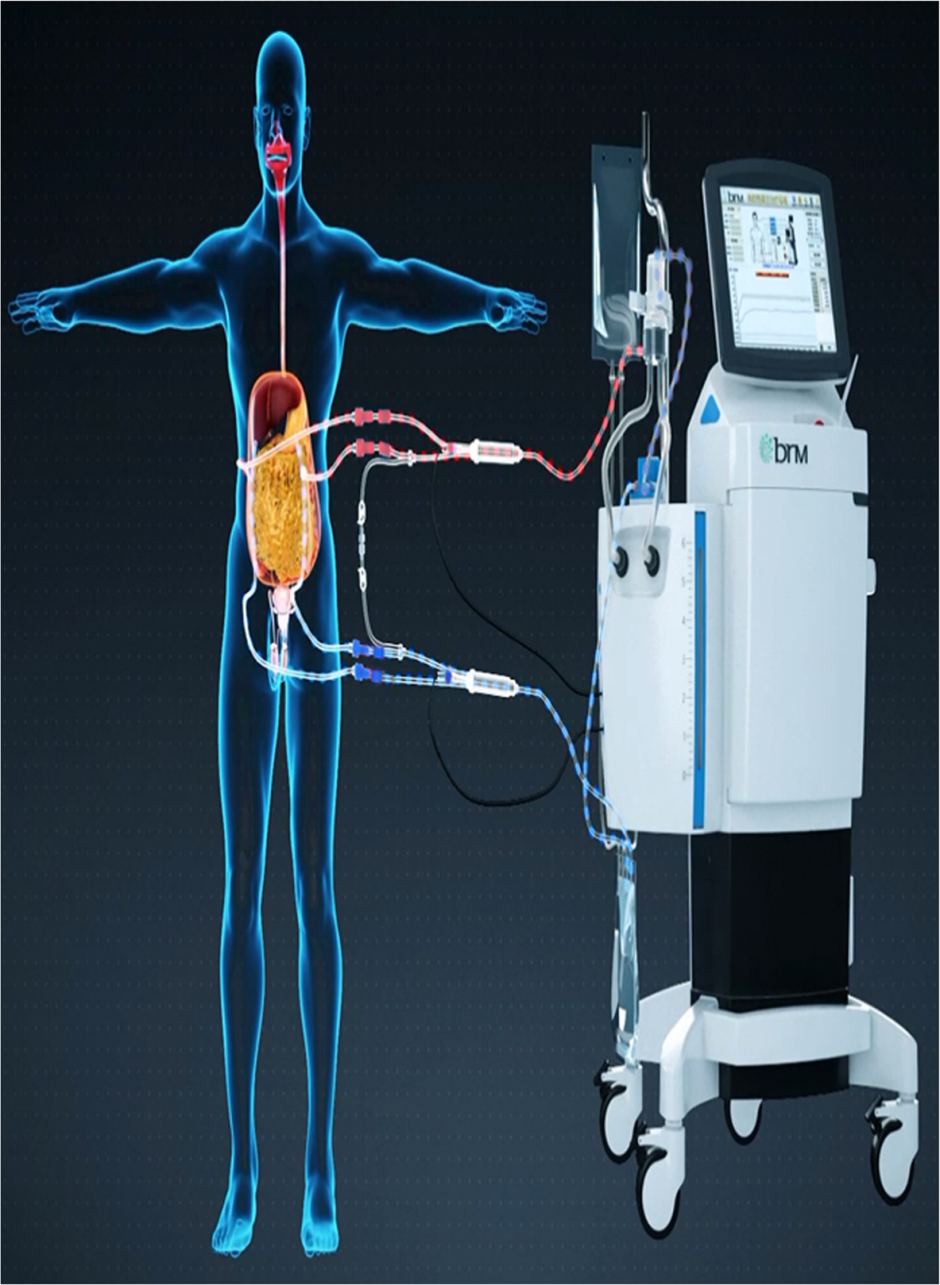
The working mechanism of intraperitoneal thermal perfusion chemotherapy. The red pipe is the external intake pipe, and the blue pipe is the external outlet pipe (Fig. 1 is cited from Guangzhou Baorui Medical Technology Co. LTD, and permission has been granted)

**Fig. 2:**
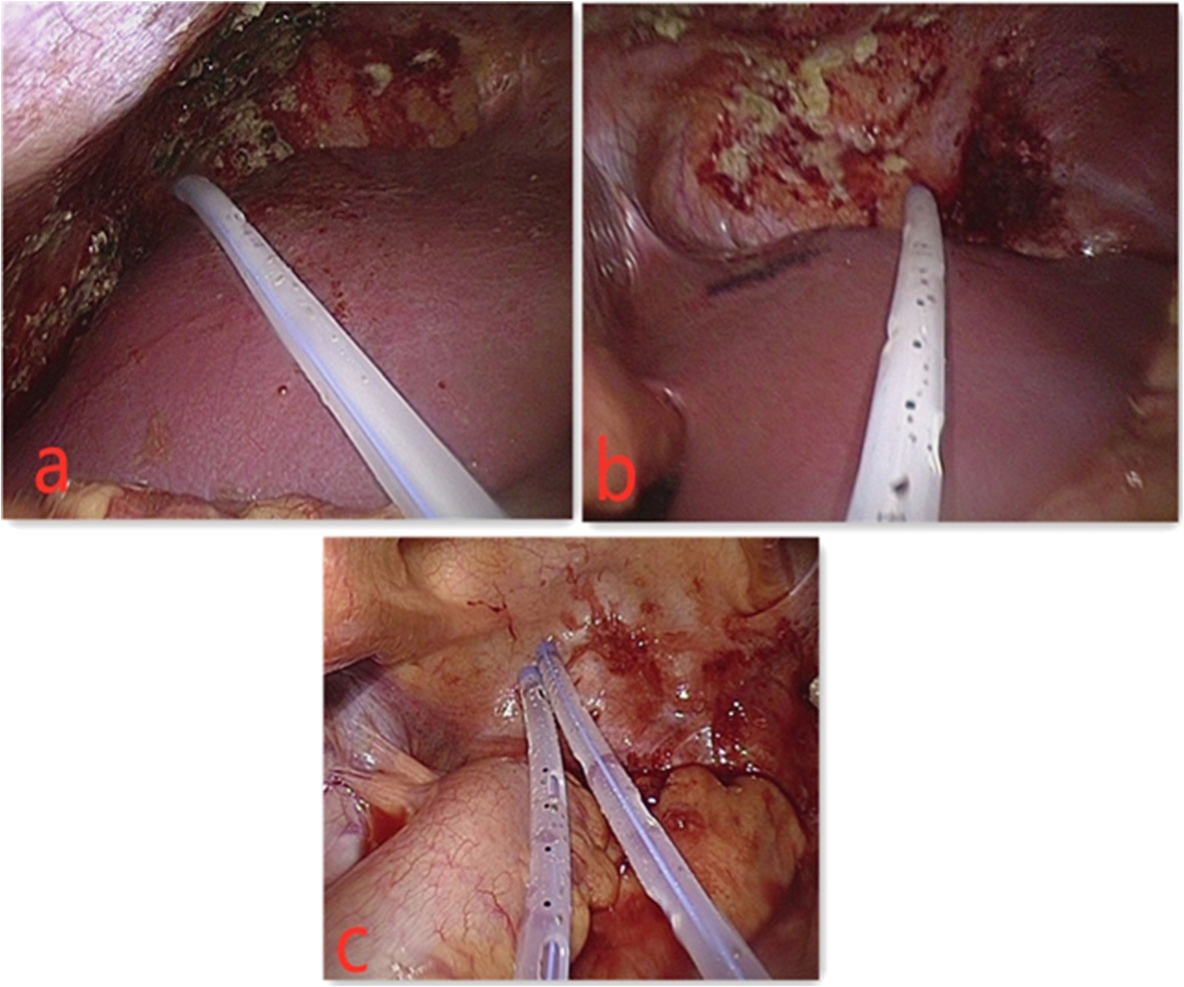
The position of the abdominal drainage tube. **a**, **b** The inlet pipe. **c** The outlet pipe

**Fig. 3:**
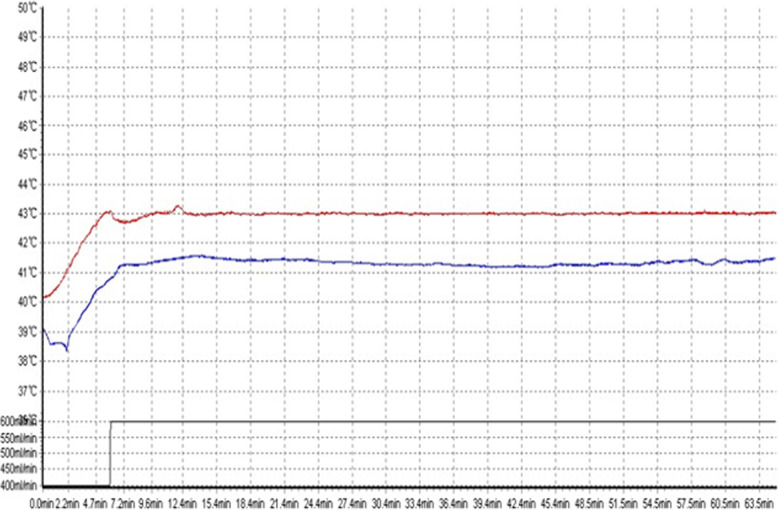
The therapeutic curve of intraperitoneal thermal perfusion. The red curve shows the inlet temperature, and the blue curve shows the outlet temperature

In the observation group, 45 patients were first treated with HIPEC for 5 times, followed by 6 ~ 8 courses of intravenous chemotherapy. The intravenous chemotherapy regimen was selected according to the primary disease [5]. The HIPEC drugs for gastric and colon cancer were loplatin, cisplatin, retetrexil, and 5-fu, while for ovarian cancer were paclitaxel, loplatin, cisplatin, and 5-fu, and the drug dosage was referred to intravenous chemotherapy [6]. The working mechanism of intraperitoneal thermal perfusion chemotherapy is shown in Fig. 1. Four special HIPEC tubes were placed under laparoscopy to connect to the HIPEC therapeutic apparatus, and chemotherapy drugs were added to 4000 ml normal saline [7]. The position of the abdominal drainage tube is shown in Fig. 2. The perfusion flow rate was 400–600 ml/min, the perfusion time was 60 min, and the treatment temperature was set as 43 ± 0.2 °C [7]. Each treatment interval was ≥ 24 h [8]. The therapeutic curve of intraperitoneal thermal perfusion is shown in Fig. 3. The HIPEC therapy instrument used in this treatment was BR-TRG-2 peritoneal thermal perfusion therapy system.

In the control group, 35 patients were treated with intravenous chemotherapy and abdominal puncture pumping. The chemotherapy regimen was selected based on the primary disease, and 6–8 courses of chemotherapy were selected. One thousand milliliters of fluid was initially dispensed, and then, no more than 500 ml of fluid was removed from the abdominal cavity every day [9]. All patients were evaluated for efficacy 1 month after the end of treatment and then followed up every month for 6 months.

### Efficacy evaluation criteria

According to WHO standards, in evaluating the clinical efficacy of patients, the patients were divided into complete remission (CR), partial remission (PR), disease stability (SD), and disease progression (PD). CR means ascites disappeared for more than 4 weeks, PR means ascites decreased by more than 50% and lasted more than 4 weeks, SD means ascites decreased by less than 50% or no change, and PD means increased ascites [10]. Total effective rate = (CR + PR)/total number of cases × 100%. Adverse reactions were classified into I ~ IV degrees according to WHO anti-cancer drug adverse reaction scale [11].

### Statistical analysis

The general clinical data and qualitative data of the treatment results of 80 patients with malignant ascites were processed by SPSS19.0 using *χ*^2^ test and quantitative data were processed by *t* test. *P* < 0.05, statistical data can be considered statistically significant.

## Result

### Vital signs and temperature of patients in the observation group during treatment

There was no significant change in vital signs and temperature in the observation group during the treatment, and the difference was not statistically significant (Table 2).

**Table 2:** Vital signs and temperature in observation group during treatment

	Treatment 0 min	Treatment 30 min	Treatment 60 min	*P* value
Heart rate (times/min)	69 ± 7	80 ± 8	94 ± 6	> 0.05
Diastolic blood pressure (mmHg)	82 ± 8	81 ± 8	94 ± 5	> 0.05
Systolic pressure (mmHg)	122 ± 6	116 ± 7	128 ± 5	> 0.05
Blood oxygen saturation (%)	98 ± 2	97 ± 2	98 ± 1	> 0.05
Respiration (times/min)	14 ± 2	16 ± 1	18 ± 1	> 0.05
Body surface temperature (°C)	36.2 ± 0.2	36.9 ± 0.3	37.7 ± 0.4	> 0.05

### Recent efficacy observations

The short-term total effective rate of patients in the observation group was 91.11%, and the short-term total effective rate of the patients in the control group was 40%. The difference between the two groups in the total effective rate shortly was statistically significant (*P* < 0.01) (Table 3).

**Table 3:** Comparison of short-term efficacy between the observation group and control group

Group	Total cases	CR	PR	SD	PD	Total effective rate
Observation group	45	13	28	2	2	91.11%
Control group	35	2	12	16	5	40.00%
*χ*^2^						25.79
*P* value						*P* < 0.01

### Adverse reactions

Both groups had varying degrees of gastrointestinal reactions, mainly vomiting, hiccups, decreased appetite, constipation, and diarrhea. Both groups had different degrees of myelosuppression, and no myelosuppression above III was found. There were no serious complications such as intestinal obstruction, intestinal adhesions, peritonitis, and abdominal infection in both groups. There was no significant difference in the incidence of adverse reactions between the two groups of patients (*P* > 0.05) (Table 4).

**Table 4:** Comparison of adverse reactions between the observation group and control group

Group	Gastrointestinal reaction			Myelosuppression	
	Grade I	Grade II	Grade III	Grade I	Grade II
Observation group	20	18	5	9	3
Control group	14	13	5	8	1
*χ*^2^	0.257			0.643	
*P* value	0.879			0.422	

## Discussion

Malignant ascites is a common complication of advanced abdominal and pelvic malignancies [12]. Abdominal puncture and fluid diuresis alone are not only ineffective, but also increase in ascites responsiveness [13]. Repeated pumping treatment is likely to lead to complications such as electrolyte disturbance and hypoproteinemi a[14]. Therefore, effectively controlling the growth of malignant ascites can significantly improve the quality of life of patients and prolong the survival of patients, which is of great significance for the treatment of advanced tumors [15]. Some scholars have pointed out that the use of hyperthermia can effectively kill tumor cells, destroy the vascular structure in the lesion area, reduce blood supply to tumor tissues, and effectively inhibit tumor proliferation [16]. In addition, hyperthermia can also effectively enhance cell membrane permeability, inhibit tumor cell self-repair, increase the permeability of drug lesions, and improve efficacy [17]. Studies have found that if the temperature of hyperthermia is lower than 41 °C, it cannot be effectively treated, but when the temperature is higher than 45 °C, normal tissues and organs will be damaged, and the critical death temperature of most tumor cells is 43 °C [18]. The temperature is controlled at 41–45 °C [19]. In this study, hyperthermia combined with HIPEC was used to intervene in the patients. Now, the perfusion chemotherapy drugs are preheated, which effectively shortens the endogenous field temperature to reach the plateau period and makes the perfusion temperature stable [20]. The results showed that the clinical efficacy of the observation group was significantly better than that of the control group and suggesting that the use of HIPEC in the treatment of malignant ascites has excellent clinical advantages.

The main purpose of treating malignant ascites is to improve the quality of life of the patient, relieve the patient’s pain, and thereby extend the patient’s survival. HIPEC is an auxiliary method used in the treatment of malignant tumors in the abdominal cavity in recent years [21]. A large number of warm chemotherapy infusion fluids can allow the chemotherapeutic drugs to fully contact the small intra-abdominal metastases. During the treatment process, the chemotherapy infusion fluid can mechanically remove free abdominal cancer cells [22]. After the chemotherapeutic drugs are infused into the abdominal cavity, there can be a constant, high, and durable concentration in the abdominal cavity, but less chemotherapy enters the systemic circulation, and the systemic adverse reactions are small, which makes HIPEC have a significant therapeutic advantage in treating malignant ascites [23].

## Conclusion

In short, intraperitoneal hyperthermic chemotherapy combined with intravenous chemotherapy can significantly control malignant ascites and has small adverse reactions, which is worthy of clinical promotion and application.

## Data Availability

The datasets during and/or analyzed during the current study are available from the corresponding author on reasonable request.
